# The utility of intraoperative endoscopy to assist novice surgeons in the detection of gastric stenosis during laparoscopic sleeve gastrectomy

**DOI:** 10.1186/s12893-022-01772-z

**Published:** 2022-08-23

**Authors:** I.-Sung Chen, Ming-Shian Tsai, Jian-Han Chen, Chung-Yen Chen, I.-Lin Chen, Chi-Ming Tai

**Affiliations:** 1 Department of Gastroenterology and Hepatology, E-Da Hospital, Kaohsiung, Taiwan; 2 Department of Surgery, Jiaan-Ren Hospital, Kaohsiung, Taiwan; 3 Bariatric and Metabolic International Surgery Center, E-Da Hospital, Kaohsiung, Taiwan; 4 School of Medicine, College of Medicine, I-Shou University, Kaohsiung, Taiwan

**Keywords:** Intra-operative endoscopy, Sleeve gastrectomy, Gastric stenosis, Leak

## Abstract

**Background:**

Laparoscopic sleeve gastrectomy (LSG) is a commonly performed bariatric surgery. Gastric stenosis and leaks are 2 major complications associated with LSG and revision surgery might be needed. Herein, we report our experience of intraoperative endoscopy (IOE) to evaluate stenosis and leaks during LSG.

**Methods:**

LSG was performed by three surgeons. Patients who underwent LSG and IOE between January 2016 and March 2020 were enrolled and assigned to two groups: group 1 (1st–30th LSG case for each surgeon) and group 2 (> 30th LSG for each surgeon). Patients’ anthropometric and biochemical data pre- and post-LSG, as well as IOE findings and follow-up esophagogastroduodenoscopy records were reviewed.

**Results:**

In total, 352 patients were enrolled including 90 patients in group 1 and 262 patients in group 2. Three out of 352 patients (0.9%) were found to have stenosis by IOE, which was related to tightly gastropexy stitch or reinforcement stitch, all of which were in group 1. Stenosis was resolved after removal of the stitch during LSG. The incidence of gastric stenosis detected by IOE was 3.3% (3/90) and 0% (0/262) in group 1 and group 2, respectively (*P* = 0.003). No leakage was found in this study and no patient developed clinical or endoscopic stenosis after LSG.

**Conclusions:**

The existing evidence showed that IOE can help detect gastric stenosis during LSG, especially for novice surgeons, and the stenosis could be resolved during operation.

## Background

The worldwide prevalence of obesity is increasing, hence it has become one of the major public health problems in both Western and Asian countries [1]. Bariatric surgery is more effective for weight loss and treating obesity-related comorbidities compared to non-operational procedures [2]. Furthermore, the demand for bariatric surgery is also increasing worldwide because of the increased prevalence of obesity [3]. Laparoscopic sleeve gastrectomy (LSG) and laparoscopic Roux-en-Y gastric bypass (LRGYB) are the two most common bariatric procedures, and both are effective at treating obesity. However, because of its technical simplicity and low complication rate, LSG is now the most common bariatric surgery [4–6].

Stenosis and leakage are two major complications of LSG, which might need revision surgery. Staple-line leak, which can cause significant morbidity and mortality, is considered the most serious complication, with a reported incidence of 0.5–2.6% [6–10] and, most frequently occurring at gastroesophageal junction [11]. The incidence of gastric stenosis is 0.6–4% and the incisura angularis affected [6, 8, 12–15]. Twisting and over-narrowing contribute to the pathogenesis of gastric stenosis which is also associated with the occurrence of gastric leak after LSG [16]. There is a higher incidence of complications with novice bariatric surgeons, which decreased as the surgeons become more experienced [17].

Intraoperative endoscopy (IOE) can be used to detect gastric stenosis and leaks during LRYGB and LSG [18–20], and is associated with a decreased risk of postoperative complications such as sepsis, unplanned reoperations, prolonged hospital stay, and composite complications after LSG and hospital stay after LRYGB [21]. The present study evaluated the routine use of IOE in LSG, particularly the occurrence of gastric stenosis and leaks in procedure performed by novice bariatric surgeons.

## Materials and methods

### Eligible patients

According to the recommendations of the Asia–Pacific consensus, patients with a body mass index (BMI) ≧ 37 kg/m^2^ or ≧ 32 kg/m^2^ with obesity-related comorbidities are recommended to lose weight to promote a healthy status and should be evaluated by a multidisciplinary team including surgeons, gastroenterologists, psychiatrists, dietitians, and endocrinologists. Patients are provided with dietary instruction, behavior modification, and pharmacologic therapy and if they fail to achieve their goal weight, bariatric surgery is suggested.

Patients were recommended to receive IOE during all bariatric procedures in our hospital. The present study is a retrospective review of the medical records of patients who underwent IOE for primary LSG from January 2016 to March 2020. Patients who did not have IOE in the LSG were excluded. Three hundred and fifty-two patients who made up more than 95% of patients receiving LSG during this period were enrolled in this study. This study was approved by our Institutional review board. (EMRP-108-072).

### Laparoscopic sleeve gastrectomy procedures

LSG was performed as detailed previously [22]. During operation, a 36-Fr orogastric tube was used as a guide during gastrectomy. After fully mobilized stomach, the sleeve gastrectomy was performed from the antrum, 5 cm away from the pylorus to the angle of His along the orogastric tube. After complete gastrectomy, we apply serosa reinforcement sutures for preventing leakage and bleeding. For preventing postoperative gastro-esophageal reflux disease, gastropexy between the gastric tube and pre-pancreatic fat was performed, with the hiatus checked regularly and the crura was closed with unabsorbable sutures if necessary. The resected portion of the stomach was removed and all wounds were closed in layers. All patients were hospitalized for 24–48 h for postoperative observation.

All procedures were performed by three surgeons (MH Tsai, JH Chen and CY Chen) experienced in laparoscopic surgery but with limited experience in LSG. The procedure was standardized in our institution. The patients were divided into subgroups according to their case number: group 1 (1st–30th LSG for each surgeon) and group 2 (> 30th LSG for each surgeon).

### Intraoperative endoscopy

With the patient in supine position, a routine IOE was performed by experienced endoscopists (CM Tai, IS Chen and IL Chen) to check for stenosis, twists, leaks, or active bleeding at the end of the sleeve procedure. Stenosis was defined when the esophagogastroduodenoscope could not reach the pylorus smoothly and stopped in the narrowest part of the sleeve. After reaching the pylorus, the endoscope was withdrawn and placed at the esophagogastric junction before the sleeve was insufflated. The surgeon submerged the sleeve in the saline and a positive leak was defined as a bubble leaking out from the sleeve. The IOE was completed when there was no gastric stenosis or leak. If stenosis was identified, the surgeon could revise it laparoscopically and recheck the sleeve by endoscopy.

### Patient follow up

Patients were scheduled follow-up 1-week, 1-month, 3-months, and 6-months after LSG. Anthropometric [weight, height, and waist circumference (WC)] and biochemical [aspartate aminotransferase (AST), alanine aminotransferase (ALT), serum cholesterol, triglyceride, high-density lipoprotein cholesterol (HDL-C), low-density lipoprotein cholesterol (LDL-C), uric acid, and glycated hemoglobin (HbA1c)] measurements were recorded before and 6 months after LSG. Esophagogastroduodenoscopy was suggested to patients to check for stenosis or other complications 6 months after LSG.

### Statistical analysis

Patients were divided into two groups. Group 1 included first 30 LSG performed by each surgeon, and group 2 contained the remaining patients. Descriptive results regarding categorical variables are presented as percentages, with continuous variables as mean and standard deviation (SD). Characteristics of these two groups were compared by using χ^2^-test and Student’s test when appropriate. Changes in clinical characteristics were compared pre- and postoperatively. Continuous variables were compared using paired t tests. A *P-*value < 0.05 indicated a statistically significant difference. All statistical analyses were performed using SPSS version 22.0 software (SPSS Inc., Chicago, IL) for Windows.

## Results

Between January 2016 and March 2020, 352 patients underwent LSG and IOE and were enrolled in this study. These patients made up more than 95% of patients who had LSG during this period in our hospital with a mean age of 35.8 years old and 47.4% were male. The mean BMI was 40.6 kg/m^2^ (Table 1). Group 1 comprised ninety patients including the first 30 LSG performed by each surgeon and group 2 contained the remaining 262 patients. There was no significant difference in clinical characteristics between group 1 and group 2 except BMI and hypertension (Table 1).

**Table 1:** Comparisons of baseline anthropometric and biochemical measurements between group 1 and group 2

Characteristics	Total n = 352	Group 1 n = 90	Group 2 n = 262	*p*
Age (years), mean (SD)	35.8 (9.7)	36.1 (10.3)	35.7 (9.5)	0.714
Male sex, *n* (%)	167 (47.4)	48 (53.3)	119 (45.4)	0.222
BMI (kg/m^2^), mean (SD)	40.6 (5.9)	39.6 (5.5)	41.0 (6.0)	0.048
Waist circumference (cm), mean (SD)	117.6 (19.5)	117.8 (14.9)	119.4 (15.0)	0.404
DM, *n* (%)	104 (29.5)	29 (32.2)	75 (28.6)	0.592
Hypertension, *n* (%)	218 (61.9)	43 (47.8)	175 (66.8)	0.004
HbA1c (%), mean (SD)	6.5 (1.6)	6.6 (1.9)	6.5 (1.4)	0.583
Total cholesterol (mg/dl), mean (SD)	199.2 (39.8)	199.9 (38.9)	198.9 (40.2)	0.844
Triglycerides (mg/dl), mean (SD)	172.3 (183.9)	186.2 (210.5)	167.4 (173.8)	0.42
HDL-C (mg/dl), mean (SD)	46.0 (10.1)	47.6 (11.2)	45.4 (9.7)	0.093
LDL-C (mg/dl), mean (SD)	121.1 (35.1)	119.6 (36.9)	121.7 (34.6)	0.630
Uric acid (mg/dL), mean (SD)	6.8 (1.8)	7.0 (1.9)	6.7 (1.8)	0.163
AST (U/L), mean (SD)	38.8 (30.9)	37.2 (27.5)	39.4 (32.0)	0.563
ALT (U/L), mean (SD)	58.8 (51.9)	58.3 (52.4)	58.9 (51.9)	0.917

*BMI* body mass index, *DM* diabetes mellitus, *HbA1c* glycated hemoglobin, *HDL-C* high density lipoprotein cholesterol, *LDL-C* low density lipoprotein cholesterol, *AST* aspartate transaminase, *ALT* alanine transaminase, *SD* standard deviation, *No.* number

### Comparisons of IOE findings between groups

Twelve patients (3.4%) received hiatal hernia repair. Three out of 352 patients (0.9%) were found to have stenosis by IOE, which was related to tightly gastropexy stitch or reinforcement stitch, all of which were in group 1. Stenosis was resolved after removal of the stitch during LSG. The incidence of gastric stenosis detected by IOE was 3.3% (3/90) and 0% (0/262) in group 1 and group 2, respectively (*P* = 0.003) but IOE leak tests were negative for all patients.

**Fig. 1:**
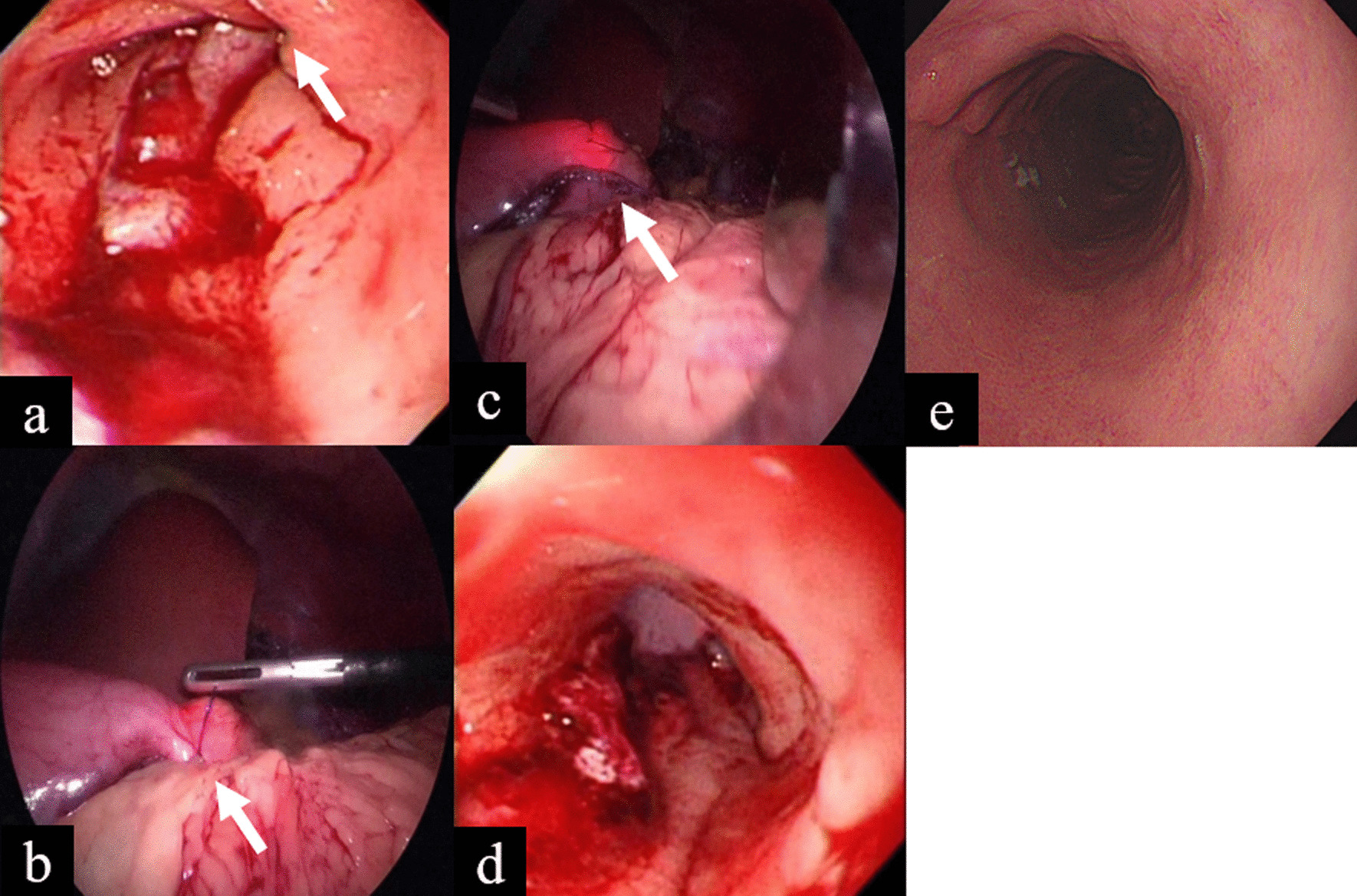
**A** Endoscopy unable to pass through the incisura angularis of the sleeve (white arrow). **B** The surgeon located the posterior stich which fixed the sleeve to the pre-pancreatic fat (white arrow). **C** The posterior stich was removed (white arrow). **D** Endoscopic view of the relieved incisura angularis. **E** Gastroscopy showed no stenosis at stomach 6 months post-surgery

As shown in Fig. 1, gastric stenosis during LSG was identified by IOE in a 39-year-old woman. She was intubated smoothly through the larynx, esophagus, gastroesophageal junction but the endoscope became stuck at the incisura angularis (Fig. 1A, white arrow). Continuous insufflation and negotiation of the sleeve failed to allow the endoscope to pass through the stenosis, so the surgeon located the tightly gastropexy stitch (Fig. 1B, white arrow) and released it (Fig. 1C, white arrow) to allow the endoscope to pass the incisura angularis smoothly to reach the pylorus (Fig. 1D). This patient underwent follow-up esophagogastroduodenoscopy 6 months after LSG and no gastric stenosis was noted during this examination (Fig. 1E).

### Effectiveness and complications after LSG

In total, 274 patients were followed up 6 months after LSG and no patients had clinical symptoms related to stenosis or leak. A comparison of clinical characteristics and endoscopic findings at baseline and 6 months after sleeve gastrectomy was performed for 101 patients with complete data available (Table 2). BMI, WC, HbA1c, triglyceride, HDL-C, uric acid, AST, and ALT all showed significant improvement after surgery. No stenosis or leak was detected by follow-up esophagogastroduodenoscopy.

**Table 2:** Comparison of clinical characteristics and endoscopic findings of 101 patients at baseline and 6 months after sleeve gastrectomy

	Baseline n = 101	6 months n = 101	*p*
BMI (kg/m^2^), mean (SD)	41.2 (6.4)	30.7 (5.5)	< 0.001
Waist circumference (cm), mean (SD)	120.3 (14.7)	97.0 (12.5)	< 0.001
HbA1c (%), mean (SD)	6.5 (1.5)	5.5 (0.4)	< 0.001
Total cholesterol (mg/dL), mean (SD)	196.2 (36.5)	188.5 (37.8)	0.075
Triglyceride (mg/dL), mean (SD)	152.4 (66.3)	86.0 (36.2)	< 0.001
HDL-C (mg/dL), mean (SD)	47.1 (9.6)	50.9 (11.3)	< 0.001
LDL-C (mg/dL), mean (SD)	119.2 (35.0)	111.7 (35.3)	0.056
Uric acid (mg/dL), mean (SD)	6.5 (1.6)	6.0 (1.6)	0.001
AST (U/L), mean (SD)	40.4 (32.4)	19.4 (5.0)	< 0.001
ALT (U/L), mean (SD)	57.9 (53.5)	18.2 (8.5)	< 0.001
Endoscopic finding			
Gastric stenosis, *n* (%)	–	0	–
Gastric leak, *n* (%)	–	0	–

*BMI* body mass index, *HbA1c* glycated hemoglobin, *HDL-C* high density lipoprotein cholesterol, *LDL-C* low density lipoprotein cholesterol, *AST* aspartate transaminase, *ALT* alanine transaminase, *SD* standard deviation

## Discussion

The present study reported the results of the routine use of IOE during LSG. No leak was detected during IOE, but 3 out of 352 patients (0.9%) were found to have gastric stenosis which was immediately relieved intraoperatively. No patients had clinical gastric stenosis or leak after LSG. Of note, all cases occurred within the first 30 cases of the surgeons starting to perform LSG suggesting that IOE can assist less experienced surgeons in LSG to prevent stenosis.

Gastric stenosis can be classified as mechanical stenosis or functional stenosis [23]. Mechanical stenosis occurs when it is difficult for the endoscope or it is unable to pass through the sleeve, whereas functional stenosis occurs when the endoscope passes through the sleeve with a variable degree of rotation. Although both types of stenosis presented similarly, the diagnosis period was different, with a mean diagnosis period for the mechanical type 9.5 days after the procedure and 43.2 days for the functional type [23]. Gastric stenosis after sleeve gastrectomy could be caused by ischemia, retraction by scarring, misalignment during stapling, prolonged operation time, or a short pylorus distance [24, 25]. In our study, the gastric stenosis resulted from tightly gastropexy stitch or reinforcement stitch and could be regarded as mechanical stenosis, which was resolved after removal of the stitch. However, if not treated, symptoms might occur soon after the operation.

Chang et al. reported that the incidence of gastric stenosis decreased from 2.1% (7/338) to 0% (0/489) after surgical standardization of LSG [8, 26]. As shown in the present study, the overall incidence of gastric stenosis detected by IOE was 0.9% (3/352). However, if the cases performed when surgeons were less experienced were excluded, no gastric stenosis occurred after LSG, indicating that care should be taken to avoid complications during this period. Although additional instruments and an experienced endoscopist are required, IOE is safe and can be used to detect complications during LSG. Nimeri et al. performed IOE during LSG and found 10 cases (3.2%) of gastric stenosis during the procedure. Stenosis resolved after removing over-sewing sutures and clinical stenosis after LSG was 0% [20]. As shown in the present study, IOE can guide the surgeons to check the cause and location of stenosis with revision performed laparoscopically.

Leaks remain the most important complication causing significant morbidity and mortality after LSG [27]. The surgeons made every effort to decrease the incidence of leaks, including omentopexy, staple-line reinforcement, oversewing suture, absorbable polymer membrane, or no reinforcement [28]. The development of a leak after LSG might result from ischemia in the gastric wall next to the staple line, stenosis of the sleeve, or increased intraluminal pressure related to low compliance of the gastric tube [11, 29]. The role of IOE in preventing leaks has been reported in several studies. Jung et al. performed a propensity-matched analysis using the MBSAQIP database and reported no significant post-LSG leaks between patients who underwent an intraoperative leak test and those who did not (0.4% vs. 0.3%, *p* = 0.05) [30]. Nimeri et al. performed IOE during LSG and reported a leak rate of 0% in primary LSG [20]. Similarly, we also detected no leaks during IOE and after the operation. It cannot be concluded suggested that IOE with a leak test did help prevent leaks after LSG, so further studies are needed to confirm this.

We reported the low percentage of stenosis and leak after LSG. Although IOE could help identify stenosis during LSG, and stenosis could be corrected during operation, the cost-effectiveness issues of IOE need to be assessed. In addition, because all stenosis occurred during the learning curve of less experienced surgeons, it is also important to evaluate whether more training with a full experienced bariatric surgeon can decrease this complication and replace IOE. Our study has several limitations. First, it was an observational study and did not include a comparative group that did not have IOE. We did not know whether the stenosis found in IOE will cause clinical symptoms if it was not relieved during LSG. Additionally, when the patient is in an upright position, the sutures hold the stomach in place, and gravity pulls the stomach and omentum down, which can cause the stomach to become acutely angled. This may not happen when the patient is in the supine position during the IOE. Second, not all patients were followed up and it is possible that some may have had signs of gastric stenosis and sought other medical help. Although complete biochemical measurement and follow-up gastroscopy were available in only 101 patients (28.7%), all patients in the present study had at least one return visit after the operation. Symptoms of gastric stenosis often occur within 3 months post-procedure [26], but none of our cohort had symptoms of gastric stenosis.

## Conclusions

In conclusion, IOE can help detect gastric stenosis during LSG, especially for surgeons less experienced in LSG. The stenosis could be corrected during the procedure, which might prevent clinical stenosis post operation. However, because no leak was detected by IOE or occurred after LSG in the present study, the use of IOE with a leak test to prevent leaks is not necessary.

## Data Availability

All data generated or analysed during this study are included in this published article.

## Abbreviations

LSG: Laparoscopic sleeve gastrectomy
IOE: Intraoperative endoscopy
